# Precise copper ion release and recovery in polycaprolactone nanofiber scaffold: an antibacterial and osteogenic synergistic strategy for guided bone regeneration

**DOI:** 10.3389/fcell.2025.1650537

**Published:** 2025-08-13

**Authors:** Tongbin Liu, Akram Hassan, Matheel Zohair Yousif Alrawas, Caiyun Cui, Zaihan Ariffin

**Affiliations:** ^1^ School of Dental Sciences, Universiti Sains Malaysia Health Campus, Kubang Kerian, Malaysia; ^2^ School of Stomatology, Qilu Medical University, Zibo City, Shandong, China; ^3^ Department of Stomatology, Binzhou Medical University Hospital, Binzhou, Shandong, China

**Keywords:** electrospinning, polycaprolactone, polydopamine, copper ions, antibacterial properties, osteogenesis

## Abstract

**Introduction:**

Infection control and bone regeneration remain major challenges in orthopedic therapy. To address these issues, we developed a multifunctional guided bone regeneration (GBR) nanofibrous material based on electrospun polycaprolactone (PCL). This material combines antibacterial and osteogenic properties using polydopamine (PDA) and copper ions (CuCu^2+^).

**Methods:**

PCL nanofibers were produced via electrospinning, and Cu^2+^ ions were introduced through PDA-mediated surface modification to enable pH-responsive binding and controlled release. The material’s physicochemical properties were evaluated through structural analysis, mechanical testing, and release kinetics. Biological performance was tested using antibacterial assays and osteoblast (MC3T3-E1) cell cultures, including assessments of cell proliferation and key osteogenic gene expression (Runx2, Osx, ALP, OCN).

**Results:**

The PCL-PDA-Cu composite showed strong structural integrity and mechanical stability. At a Cu^2+^ concentration of 0.1 M, it demonstrated: 1) strong antibacterial activity; 2) improved osteoblast proliferation; and 3) increased expression of osteogenic genes. The pH-dependent release system maintained effective Cu^2+^ levels while reducing cytotoxicity.

**Discussion:**

By integrating PDA-mediated Cu^2+^ coordination with PCL nanofibers, we created a multifunctional platform that balances antimicrobial defense and bone regeneration. This controlled ion delivery strategy shows great promise for bone tissue engineering, especially in infection-prone environments.

## 1 Introduction

Electrospinning technology, with its ability to create fibers spanning the micron-to-nanometer range, has emerged as a strong tool in biomedical materials (Xue et al., 2019; Tardy et al., 2021). Polycaprolactone (PCL)-based electrospinning nanofibers have attracted significant attention because of their excellent mechanical properties and highly adjustable three-dimensional porous structures that closely resemble the extracellular matrix (ECM). These characteristics render them exceptionally appropriate as scaffolds for tissue engineering and regenerative medicine (Dutcher, 2018). Despite these advantages, PCL nanofibers exhibit inadequate hydrophilicity and a chemically inert surface, which significantly hinders their ability to support cell adhesion, proliferation, and tissue repair. To mitigate this limitation, polydopamine (PDA) coatings have been implemented as a biomimetic technique. Owing to their rich phenolic hydroxyl (-OH) and other functional groups. PDA coatings can create a durable modification layer on the fiber surface, markedly enhancing its wettability and biocompatibility (Tan and Zhou, 2018; Yang et al., 2021). Beyond enhancing hydrophilicity, PDA coatings offer versatile functionalization sites, allowing the nanofibers to integrate antimicrobial agents, metal ions, including copper ions, or growth factors. This multifunctionalization expands the biological applications of PCL nanofibers and opens new avenues for developing advanced biomedical materials. This composite material design effectively addresses the inherent limitations of PCL by leveraging PDA’s ability to modify surface properties and enhance bioactivity while offering a promising platform for next-generation biomedical applications (Xie et al., 2021).

Due to their dual biological activity, especially in antibacterial and bone regeneration applications, copper ions have attracted the interest of academia. Their antimicrobial efficacy stems from various mechanisms, including bacterial membrane disruption, interference with metabolic pathways, and the induction of reactive oxygen species (ROS) generation (Xia et al., 2023; You et al., 2022), rendering them highly effective against a broad spectrum of pathogens. Simultaneously, copper ions are essential in bone regeneration by acting as cofactors for key enzymes and cytokines, significantly enhancing osteoblast adhesion, proliferation, and differentiation (Sun et al., 2024). The biological effects of copper ions are highly concentration-dependent. Excessive copper ion concentration can induce oxidative stress and cytotoxicity in normal cells and tissues, while insufficient concentrations fail to provide adequate antimicrobial protection or support for bone formation (Chen Y. et al., 2024). Therefore, precise control over copper ion release is essential for maintaining a delicate balance between its antimicrobial properties and bone regeneration capacity. This study introduces a novel approach utilizing a PDA coating to regulate copper ion release according to environmental conditions.

**FIGURE 1:**
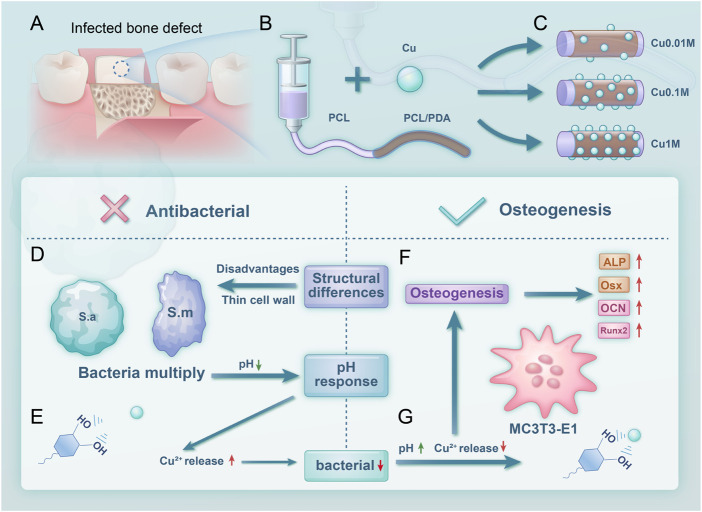
Scheme 1 Experimental design and process. **(A)** Material application in treating infectious bone defects; **(B)** Material synthesis process; **(C)** Grouping of the material; **(D)** Antibacterial mechanism; **(E)** Copper ion release increases at lower pH; **(F)** Osteogenic performance; **(G)** Copper ion release decreases at higher pH.

The catechol groups in the PDA structure form stable chelation bonds with copper ions, providing the system with an effective and adjustable release mechanism. In acidic conditions, these chelation bonds weaken, facilitating the release of copper ions, increasing their local concentration and enhancing antimicrobial effectiveness (Xiao et al., 2023). As the concentration of Cu^2+^ increases, copper exists primarily in the form of ${\text{Cu}}^{2+}$ and CuO, while the phenolic hydroxyl (-C-OH) groups in PDA gradually oxidize into quinone (-C-O-) structures. Simultaneously, the protonation level of pyrrole nitrogen increases, signifying that copper ion binding alters its own chemical state and induces structural transformations in PDA, which subsequently affect the material’s surface properties and biological activity. Once the infection is controlled and the local pH returns to neutral, the PDA coating effectively re-sequesters free copper ions, thereby preventing excessive diffusion into surrounding tissues and mitigating potential toxicity (Chang et al., 2024; Han et al., 2024). This dynamic release-and-recapture strategy ensures a fine-tuned balance between antimicrobial efficacy and tissue compatibility and enhances the overall safety and multifunctionality of the material. This study successfully develops a PCL nanofiber composite with integrated antimicrobial and bone regenration properties. This advancement significantly improves biocompatibility and application safety, providing a promising direction for developing next-generation bio-functional materials while mitigating the longstanding biosafety concerns of previously used antimicrobial systems (Figure 1).

## 2 Materials and methods

### 2.1 Electrospinning nanofiber scaffolds’ fabrication and preparation

We dissolved 1 g of PCL in a mixture of 4 mL dichloromethane (DCM) and 3 mL N, N-dimethylformamide (DMF), and stirred the mixture continuously for at least 8 h to obtain a homogeneous suspension. After allowing the solution to stand for 15 min, we transferred it into a syringe. Electrospinning was performed using a micro syringe pump (SN-F8T, China) at a flow rate of 3 mL/h, with a 23-gauge needle, an applied voltage of 25 kV, and a collector distance of 20 cm.

### 2.2 PCL-PDA’s fabrication and preparation

(1) Dopamine Hydrochloride Solution’s fabrication and preparation. We dissolved 1.21 g of Tris in 800 mL of deionized water and stirred until they became fully dissolved. Subsequently, we gradually added hydrochloric acid (HCL) to adjust the pH to 8.5; thereafter, the final volume was adjusted to 1,000 mL with deionized water. We added 10 mL of the prepared Tris-HCl buffer solution and dissolve 20 mg of dopamine hydrochloride powder using ultrasonic assistance to obtain a 2 mg/mL dopamine hydrochloride Tris-HCl buffer solution. The prepared solution was reserved for further use. (2) We submerged the electrospinning PCL scaffold into the prepared dopamine solution and incubate it for 4 h on a shaker to facilitate dopamine polymerization. After incubation, we rinsed the scaffold with deionized water to remove unbound PDA, followed by vacuum drying for 24 h at room temperature.

### 2.3 PCL-PDA-cu’s fabrication and preparation

We dissolved 0.0135, 0.135, and 1.35 g of ${\text{CuCl}}_{2}$ powder into 10 mL of the 2 mg/mL dopamine-Tris-HCl buffer solution to obtain ${\text{CuCl}}_{2}$ solutions with concentrations of 0.01, 0.1, and 1 mol/L, respectively. We immersed the electrospinning scaffold in the dopamine- ${\text{CuCl}}_{2}$ mixed solution and incubate it for 4 h to allow copper ion loading. The treated scaffolds were subsequently vacuum-dried at room temperature for future use. Before cell seeding, all steps were performed under sterile conditions, and the scaffolds were exposed to UV light for 4 h to ensure sterility.

### 2.4 Scaffolds morphology and elements distribution

Before testing, all samples were desiccated at room temperature to remove any potential moisture. Electrospinning scaffolds—PCL, PCL-PDA, and PCL-PDA-Cu—were sectioned into appropriate sizes and mounted on sample holders using conductive adhesive. A field-emission scanning electron microscope (FE-SEM, Tescan MIRA, Czech Republic) was utilized to examine the surface morphology, including structural features, pore dimensions, fiber diameter, and surface roughness. Energy-dispersive X-ray spectroscopy (EDS) was employed to analyze the chemical composition of the scaffold surfaces, and confirmed the presence of copper ions and the PDA coating. X-ray photoelectron spectroscopy (XPS, Thermo Fisher ESCALAB 250Xi, USA) was performed to examine copper ion loading and its interactions with the PDA coating. XPS data provided insights into alterations in surface functional groups, supporting the role of copper ions in promoting PDA chemical transformation.

### 2.5 Determine of optimal concentration of copper ion loading

This study initially estimated the effect of different copper ion concentrations on MC3T3-E1 bone regrowth cells using Cell-Counting Kit-8 (CCK-8) assay to determine the optimal concentration of copper ion loading. Cells were nurtured on PCL-PDA-Cu scaffolds with varying concentrations of copper ions before their metabolic activity was determined using CCK-8 kits.

### 2.6 Copper ion release

The study immersed 2cm×2cm (approximately 10 mg) electrospinning PCL-PDA-Cu membranes in 5 mL of phosphate-buffered saline (PBS) and placed them in 15 mL centrifuge tubes. The tubes were incubated in a temperature-controlled shaker (37°C, 100 rpm/min). The immersion duration was as follows: 1, 2, 4, 6, 8, 10, 12, 14, 16, 18, 20, and 22 days. The solution was refreshed, and samples were collected. Inductively coupled plasma mass spectrometry (ICP-MS, Thermo ICP MS iCAPQ, ThermoFisher, USA) was employed to assess the copper ion concentration in PBS. Each condition was tested and analyzed in triplicate.

### 2.7 Tensile strength

The electrospinning scaffolds, including PCL, PCL-PDA, and PCL-PDA-Cu, were cut into rectangular strips measuring 5 cm n length and 3 cm in width. At room temperature, the study employed a universal testing machine to measure the tensile strength at a tensile speed of 10 mm/min. Each scaffold selected at least three samples. The yield point of the stress-strain curve is the tensile strength. The slope of the linear part represents the elastic modulus. At last, the average value was calculated.

### 2.8 Water vapor transmission rate (WVTR)

The study evaluated their ability to regulate humidity based on the ASTM standard tested nanofibers’ water vapor transmission rate (WVTR).

$$WVTR=\frac{G}{T\;\times \;A}$$

### 2.9 Water contact angle

A water contact angle (WCA) goniometer (SZ-CAMC33, China) was utilized to assess the hydrophilicity of various scaffold samples. Water droplets were placed in six different positions of each sample to statistically analyze the surface wettability and evaluate its effect on cell adhesion.

### 2.10 Surface roughness detection

The samples were placed on a white-light interferometer platform (RTEC LAMBDA, USA), utilizing the white-light interferometry technology to scan the fiber surface and generate a 3D surface profile.

### 2.11 Material degradation and weight loss

A weight loss experiment was conducted to examine material degradation under simulated physiological conditions. Morphological changes before and after degradation were observed using field emission scanning electron microscopy (FE-SEM).

### 2.12 Cell culture

The MC3T3E1 preosteoblast cell line (mouse calvaria origin) was obtained from Shanghai Zhong Qiao Xin Zhou Biotechnology Company. The cells were nurtured in a complete αMEM medium supplemented with 10% fetal bovine serum (FBS) and 1% penicillin-streptomycin. They were incubated at 37° C, a humidity rate of 95%, and a ${\text{CO}}_{2}$ concentration and 5% and were confirmed to be mycoplasma-free using polymerase chain reaction (PCR) testing. The media were replaced every 2 days. Cells were removed using 2.5 g/L trypsin, centrifuged, and transferred to new culture flasks when confluence reached 80%–90%. The experimental cell passage is within ten. Before the cell seeding, electrospinning scaffolds were sectioned into 15 mm diameter discs to fit 24-well plates and subjected to UV irradiation for 2 h to ensure both sides were exposed. They were fixed to 24-well plates utilizing sterilized stainless-steel rings and immersed in a complete medium containing 1% antibiotics for 1 h to enhance sterilization and hydration.

### 2.13 Cytoskeleton staining

The cytoskeleton staining experiment was performed to assess the morphology and differentiation of MC3T3-E1 osteoblasts cultured on PCL-PDA-Cu scaffolds. Initially, MC3T3-E1 cells were seeded onto the PCL-PDA-Cu scaffold surface containing different copper ion concentrations and incubated for 24 h. After incubation, cells were fixed with 4% paraformaldehyde and subsequently rinsed with PBS. The cytoskeleton was stained with FITC-labeled anti- α -tubulin to visualize microtubules and phalloidin-TRITC to stain actin filaments. The stained samples were subsequently examined under a fluorescence microscope to assess cytoskeletal architecture and cell morphology.

### 2.14 Live/dead cell staining

Osteoblasts were seeded onto PCL, PCL-PDA, and PCL-PDA-Cu electrospinning scaffolds as described above. After 12 h, the 24-well plate was collected, and cell viability was evaluated using the Calcein-AM/PI dual-staining kit. Fluorescence microscopy was employed to observe live and dead cells adhered to the scaffold surfaces, offering insights into the biocompatibility of the scaffold materials. The experiment was repeated thrice, and for each sample, five regions (top, middle, bottom, left, and right) were examined to ensure data reliability.

### 2.15 Alkaline phosphatase (ALP) measurement

ALP activity is a key indicator for evaluating the early differentiation of osteoblasts. As described above. Osteoblasts were seeded onto PCL, PCL-PDA, and PCL-PDA-Cu electrospinning scaffolds and cultured for 1 day. The medium was subsequently replaced with a bone regrowth induction medium (complete medium supplemented with 0.05 g/L vitamin C, 10 mM β -glycerophosphate, and 0.1 μ M dexamethasone) for continued culture over 7 days. After incubation, the 24-well plate was collected, and ALP staining was performed using a BCIP/NBT Alkaline Phosphatase Color Development Kit. The staining results were observed under an inverted microscope to assess bone regrowth differentiation. The experiment was repeated thrice, and for each sample, five regions (top, middle, bottom, left, and right) were analyzed to ensure data reliability.

### 2.16 Alizarin red S (ARS) measurement

Osteoblasts were seeded onto PCL, PCL-PDA, and PCL-PDA-Cu electrospinning scaffolds were cultured for 1 day. The medium was subsequently replaced with bone regrowth induction medium (complete medium supplemented with 50 μ M vitamin C, 10 mM β -glycerophosphate, and 0.1 μ M dexamethasone) and cultured for 21 days. Following incubation, the 24-well plate was collected, and ARS staining was performed to visualize calcium nodule formation.

### 2.17 Antibacterial test

This study selected two common oral microbes, *Staphylococcus aureus* (ATCC 25923) and *Streptococcus* mutans (UA159), to partially verify the antibacterial properties of the electrospinning scaffolds. These bacterial strains were obtained from the Department of Microbiology, Binzhou Medical University Affiliated Hospital. Both strains were cultivated in Brain Heart Infusion (BHI) medium. The medium was sterilized at 121 ° C for 15 min using autoclaving. Liquid media were used after cooling, while solid media were poured into Petri dishes (15–20 mL per dish) at 50°C and allowed to solidify for 15 min before storage.

#### 2.17.1 Bacterial inoculation

A sterilized inoculation loop was utilized to transfer single colonies of *S. aureus* (*S. aureus*) *S. aureus* and *Streptococcus* mutans (S. mutans) into separate bacterial culture tubes containing BHI liquid medium. The cultures were incubated at 37 ° C with shaking (200 rpm) for 24 h.

#### 2.17.2 Bacterial concentration measurement

The bacterial concentration was determined using a Merck Densicheck electronic turbidity meter. The bacterial suspension was diluted with 0.85% physiological saline to a final concentration of $10^{6}$ CFU/mL. Turbidimetry was utilized to indirectly estimate bacterial density by measuring light transmittance. Bacterial concentration is inversely proportional to transmittance and directly proportional to optical density within a specific range. This method is convenient and efficient.

#### 2.17.3 Antibacterial rate

Electrospinning scaffolds were pre-treated and sterilized as previously described. Sterile electrospinning scaffolds (15 mm in diameter) were co-incubated with 1.5 mL of $10^{6}$ CFU/mL *S. aureus* or S. mutans at 37 ° C incubator for 24 h. Subsequently, each sample was removed from the bacterial suspension and placed into 1 mL of 0.85% physiological saline. The mixture was vortexed for 3 min to detach bacteria from the scaffold. The bacterial suspensions were subsequently serially diluted, and 100 μ L of each dilution was spread onto BHI solid medium plates using a streaking method. After incubation at 37 ° C incubation for 24 h, bacterial colonies were counted using the plate counting method to calculate antibacterial efficiency: Antibacterial rate (%) = (control group–experimental group)/control group  ×  100.

#### 2.17.4 Live/dead bacterial staining

Electrospinning scaffolds (15 mm in diameter) were co-incubated with 1 mL of $10^{6}$ CFU/mL *S. aureus* or S. mutans at 37° C for 24 h. After incubation, each sample was removed from the 24-well plate and gently rinsed with 0.85% physiological saline. Bacterial viability was evaluated using the ${\text{MycoLight}}^{\text{TM}}$ Fluorescent Live/Dead Bacterial Staining Kit. Staining was performed according to the manufacturer’s protocol, and bacterial adhesion to the scaffold surface was examined using fluorescence microscope. The experiment was repeated thrice, and for each sample, five regions (top, middle, bottom, left, and right) were examined to ensure data reliability.

#### 2.17.5 SEM observation of bacteria on scaffold surfaces

Electrospinning scaffolds (15 mm in diameter) were co-incubated with 1 mL of 10^6^ CFU/mL *S. aureus* or S. mutans at 37° C for 24 h. After incubation, each sample was removed from the 24-well plate and rinsed with 0.85% physiological saline and fixed with 2.5% glutaraldehyde for 4 h. A graded ethanol series (10%, 30%, 50%, 70%, 90%, 95%, 100%) was used for dehydration every 15 min after air drying; the samples were examined as soon as possible using SEM to observe bacterial morphology on the scaffold surface.

#### 2.17.6 Bacterial intracellular protein leakage measurement

After the three bacterial groups were treated, they were centrifuged, and the supernatant was collected into a 96-well plate. The BCA protein assay kit (Beyotime, P0012S) was used to determine protein leakage. The samples were incubated in the dark for 30 min, and optical density (OD) at 562 nm was measured using a microplate reader (Biotek Synergy 2, USA).

#### 2.17.7 Antibacterial zone test

The bacterial media of the three treated groups were inoculated onto the surface of agar plates using a sterile cotton swab to ensure uniform distribution. Sterile filter paper discs containing antimicrobial agents were subsequently placed on the agar surface with controlled drug concentrations. After incubation, the diameter of the inhibition zones surrounding each disc was measured to assess the antibacterial efficacy.

### 2.18 Statistical analysis

Data are expressed as mean ± standard deviation. GraphPad Prism software was used to perform statistical analysis. One-way analysis of variance was applied for group comparison. A p<0.05 was considered statistically significant.

## 3 Results and discussions

### 3.1 Analysis of surface morphology of electrospinning membranes

**FIGURE 2:**
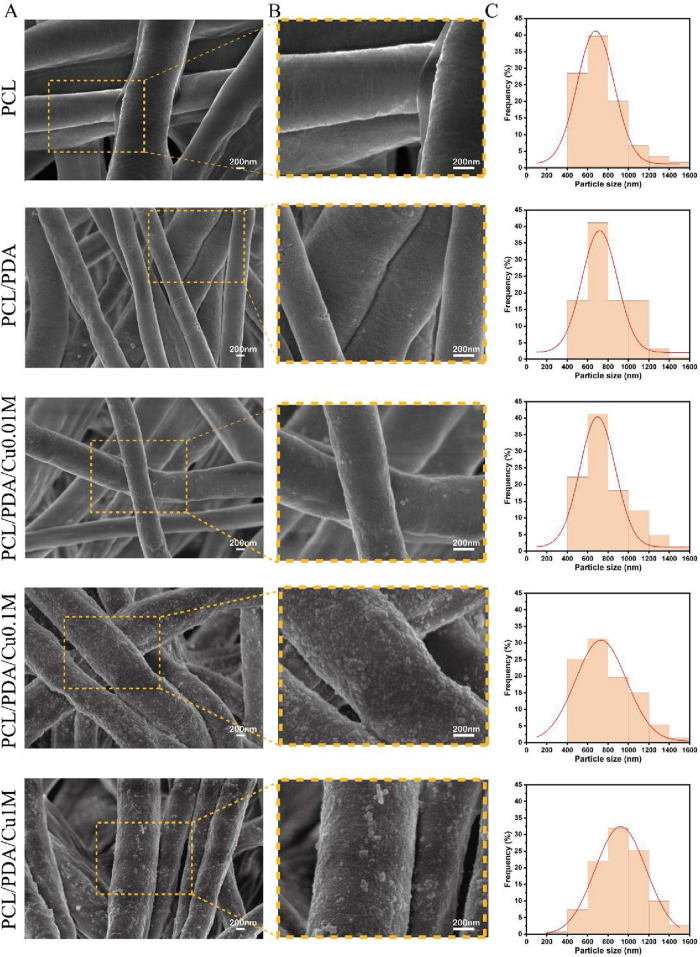
Morphology and size distribution of electrospinning membranes. **(A,B)** SEM image of electrospinning membranes. **(C)** Fiber Diameter Stats.

The morphology of the electrospinning scaffolds was examined using SEM (Figure 2). This study successfully produced five groups of electrospinning nanofiber scaffolds with uniform fiber diameters and continuous, smooth fiber surfaces. The fibers were arranged randomly, creating a porous three-dimensional network structure. Fiber diameter analysis using ImageJ software revealed that the average diameter of unmodified PCL fibers was approximately 678.5±18.9nm . After PDA coating, the fiber diameter significantly increased to 717.4±32.8nm , accompanied by an increase in surface roughness, signifying the successful deposition of PDA on the scaffold surface. Further observations revealed that PCL-PDA-Cu scaffolds exhibited significant particle aggregation on the fiber surface, probably due to the co-deposition of copper ions with PDA (Xu H. et al., 2023). The co-deposition action altered the fiber morphology and enhanced the antibacterial and bone regrowth properties of the scaffold (Chen M. et al., 2024). Despite the significant structural modifications, the porous structure and overall framework stability of the three-dimensional scaffold were effectively preserved, which is essential in bone tissue engineering.

### 3.2 Mapping and XPS results

**FIGURE 3:**
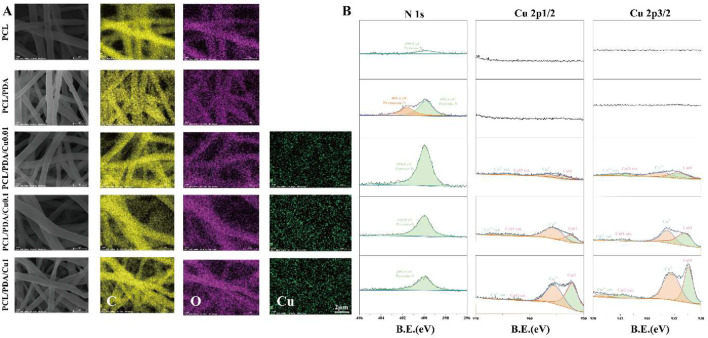
**(A)** EDS diagram of electrospinning membranes; **(B)** X-ray photoelectron spectroscopy of electrospinning membranes.

Elemental mapping (Figure 3A) indicated that copper ion distribution within the PCL/PDA composite increased with rising ${\text{Cu}}^{2+}$ concentration. In the Cu1 group (0.01M ${\text{Cu}}^{2+}$ concentration), weak and sparsely distributed copper signals were observed, indicating low ${\text{Cu}}^{2+}$ release and minimal aggregation in the material. In the Cu2 group (0.1M ${\text{Cu}}^{2+}$ concentration), there are moderate and well-distributed copper signals, indicating sufficient ${\text{Cu}}^{2+}$ release and uniform integration within the material. The Cu3 group (1M ${\text{Cu}}^{2+}$ concentration) exhibited the strongest and most uniformly distributed copper signals, with a ${\text{Cu}}^{2+}$ mass fraction of 3.24 ± 0.39%, indicating extensive copper ion aggregation on the surface and interior of the scaffold. These findings confirm that ${\text{Cu}}^{2+}$ concentration directly affects its distribution and release, with elevated ${\text{Cu}}^{2+}$ levels resulting in increased surface incorporation, potentially impacting the scaffold’s biological properties (Zhu et al., 2021).

XPS analysis (Figure 3B) revealed the impact of varying ${\text{Cu}}^{2+}$ concentrations (0.01, 0.1, and 1 M) on the copper compounds and chemical structure of PDA on the PCL/PDA surface. In the Cu2p region, distinctive peaks of ${\text{Cu}}^{2+}$ and CuO appeared in the Cu2p3/2 and Cu2p1/2 regions, with binding energies of 934.7 and 932.6 eV, as well as 955.1 and 953.6 eV, respectively. Additionally, their satellite peaks were located at 944.1 and 941.2 eV, as well as 963.7, and 961.6 eV. As the ${\text{Cu}}^{2+}$ concentration increased, the chemical state of copper remained unchanged, existing primarily as ${\text{Cu}}^{2+}$ and CuO. In the O1s region, with increasing ${\text{Cu}}^{2+}$ concentration, the -C-OH groups in PDA gradually transformed into quinone (-C-O-) structures. In Cu1-PDA-PCL, C-OH groups constituted 61.4%, while quinone structures comprised 38.6%. In Cu3-PDA-PCL, C-OH groups decreased to 32.6%, while quinone structures increased to 67.4%. Furthermore, N1s region analysis indicated that as ${\text{Cu}}^{2+}$ concentration increased, pyrrolic nitrogen was transformed into protonated nitrogen. At high ${\text{Cu}}^{2+}$ concentration (62.5 mg/mL), the percentage of protonated nitrogen reached 57.4%. These alterations indicate that ${\text{Cu}}^{2+}$ chelation promoted the conversion of C-OH groups to quinone and accelerated pyrrolic nitrogen protonation (Yi et al., 2023). Overall, the introduction of ${\text{Cu}}^{2+}$ altered the chemical state of copper and facilitated the chemical transformation of PDA through chelation, potentially affecting the material’s properties and applications.

### 3.3 Optimal copper ion concentration selection

**FIGURE 4:**
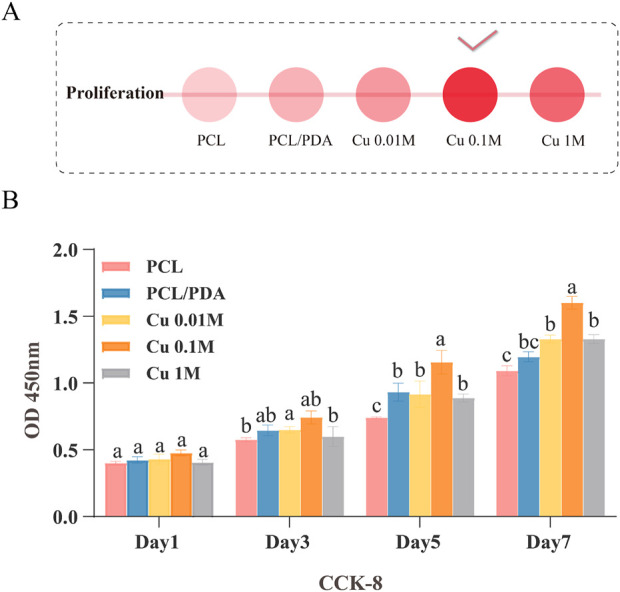
Screening of the optimal copper ion concentration in the material. **(A)** Description of panel A, if available. **(B)** CCK-8 assay evaluating the effect of different copper ion concentrations on MC3T3-E1 cell proliferation.

Herein, we investigated the effects of different copper ion concentrations on the cell proliferation of PCL/PDA composite materials. First, the CCK-8 assay was utilized to evaluate the effect of different copper ion concentrations on the MC3T3-E1 cell proliferation (Figures 4A,B). Experimental results demonstrated that cell proliferation was most significant when the copper ion concentration was 0.1 M. Compared to the control group, the cell proliferation rate was significantly increased ( p<0.001 ). Specific data indicated that under 0.1 M copper ion treatment, the cell proliferation rate increased by approximately 40.7%±7.1% . This concentration demonstrated the optimal cell proliferation effect and exhibited good biocompatibility and safety in biological tests (Du et al., 2022; Geng et al., 2024). Considering cell proliferation and biocompatibility, 0.1 M copper ion concentration was selected as the optimal concentration for the experiments (Qi et al., 2022).

### 3.4 Material synthesis and characteristics

#### 3.4.1 Thermogravimetry

**FIGURE 5:**
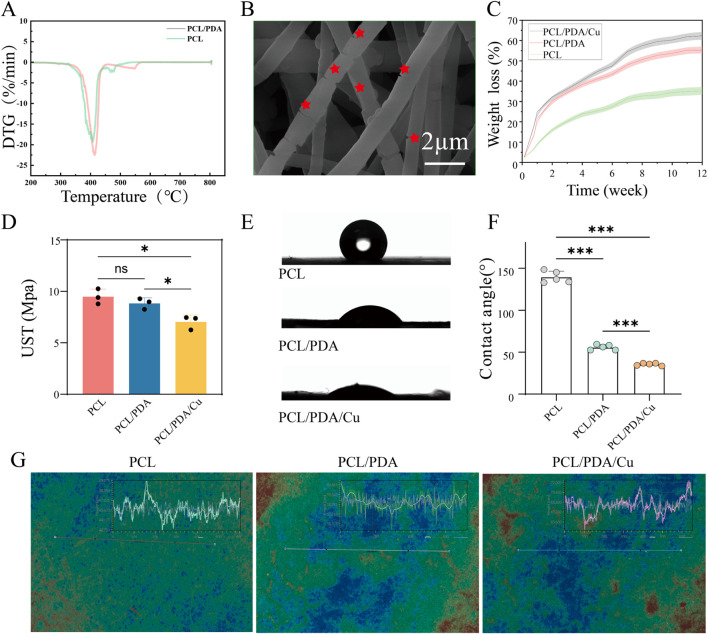
Characterization of fiber membranes. **(A)** Thermogravimetric analysis of fiber membranes in each group. **(B)** Morphological changes after degradation were observed using FE-SEM. **(C)** The weight loss rate of fiber membranes in each group was calculated using the weight loss method. **(D)** Ultimate tensile strength analysis of fiber membranes in each group. **(E, F)** Hydrophilicity analysis of fiber membranes in each group. **(G)** Characteristic images obtained from white light interferometry.

Thermogravimetric analysis (Figure 5A) revealed significant differences in thermal stability between PCL and PCL/PDA composites. For pure PCL, the thermal degradation process began at approximately 300° C, and the maximum degradation rate occurred at approximately 340°C, which revealed that the phenomenon is primarily due to polyester chain breakage, the minimal weight loss was before 450° C, indicating excellent thermal stability (Sanjarnia et al., 2023). However, PCL/PDA composites’ early weight loss occurred at 100−150° C, primarily due to water evaporation and low-molecular-weight PDA compound volatilization. As temperature increases, a second significant weight loss appeared at ∼350° C, which indicated that PDA degradation contributed to the overall decomposition process (Zhao et al., 2023). PCL/PDA composites exhibited a slightly lower degradation onset temperature than pure PCL, accompanied by significant mass loss during thermal degradation. This indicates that PDA marginally diminished the thermal stability of the composite. However, this thermal modification may be beneficial for applications requiring tunable degradation properties, especially in mild-temperature or biomedical environments.

#### 3.4.2 Other material characteristics

##### 3.4.2.1 Weight loss

Results and Discussions: Figure 5H, depicts that 30-day weight loss test, the degradation behavior of PCL, PCL/PDA, and PCL/PDA/Cu scaffolds exhibited certain differences. Pure PCL’s weight loss is minimal, indicating high stability. However, PCL/PDA and PCL/PDA/Cu exhibited significantly increased weight loss. The differences between these two groups were negligible, suggesting that PDA exerted a dominant role in degradation. PDA degradation is probably due to the hydrolysis and cleavage of phenolic -OH and quinone groups within its structure, making PCL/PDA and PCL/PDA/Cu more susceptible to hydrolytic degradation in aqueous environments (Yan et al., 2024). These results are consistent with those of previous reports on PDA-based composites, including the work by Zhang et al. (2022), who reported that PDA coatings significantly enhanced hydrolysis rates (Zhang et al., 2022). Although ${\text{Cu}}^{2+}$ incorporation may influence degradation rates under specific conditions, herein, PDA-induced degradation dominated the overall weight loss process. Statistical analysis confirmed that PCL/PDA and PCL/PDA/Cu scaffolds exhibited significantly higher weight loss than pure PCL (P < 0.05), but no significant difference was observed between PCL/PDA and PCL/PDA/Cu (Kim et al., 2023).

##### 3.4.2.2 Tensile stress

Results and Discussions: Figure 5F depicts that during the tensile stress test, PCL, PCL/PDA, and PCL/PDA/Cu exhibited various mechanisms. Pure PCL’s high tensile strength was 9.23±1.05 MPa with good ductility, indicating strong mechanical integrity. PCL/PDA’s tensile stress decreased to 8.15±0.78 MPa, probably due to microstructural changes induced by PDA incorporation. PDA, as a flexible material, may reduce intermolecular interactions between polymer chains, compromising tensile properties (Qi et al., 2023). The tensile strength of PCL/PDA/ ${Cu}^{2+}$ was significantly lower than that of PCL/PDA, suggesting that ${\text{Cu}}^{2+}$ incorporation severely affected the mechanical properties of the nanofibers. Previous studies, including those of Kan et al., have reported that PDA coatings enhance degradability and hydrophilicity but perhaps at the expense of mechanical performance (Kan et al., 2025). Statistical analysis confirmed that PCL/PDA/Cu exhibited significantly lower tensile strength than pure PCL (p<0.05) (Yuan et al., 2021) (Figure 5G).

##### 3.4.2.3 Water vapor transmission rate (WVTR)

Results and Discussions: Figure 5C depicts that during the WVTR test. PCL, PCL/PDA, and PCL/PDA/Cu, exhibited different water vapor permeability. Pure PCL exhibited a low WVTR (around 875 g $\cdot {\text{m}}^{-2}\cdot {\text{d}}^{-1}$ ), indicating strong barrier properties, making it suitable for applications requiring high moisture resistance (Liu et al., 2025). However, PCL/PDA exhibited a significant increase in WVTR (about 1,589 g $\cdot {\text{m}}^{-2}\cdot {\text{d}}^{-1}$ ), probably because of enhanced hydrophilicity from PDA incorporation, which facilitated water vapor permeation. PCL/PDA/Cu exhibited a WVTR of approximately 1,487 g $\cdot {\text{m}}^{-2}\cdot {\text{d}}^{-1}$ , slightly higher than PCL but similar to PCL/PDA, indicating that ${\text{Cu}}^{2+}$ exhibited little effect on WVTR. Overall, PCL/PDA and PCL/PDA/Cu scaffolds exhibited significantly higher WVTR than pure PCL, indicating improved moisture permeability, which is beneficial for wound dressing applications. Statistical analysis revealed that PCL/PDA and PCL/PDA/Cu exhibited significantly higher WVTR than pure PCL ( p<0.05 ) (An et al., 2024).

##### 3.4.2.4 Water contact angle measurement

The water contact angle test confirmed the effect of dopamine and copper ions on the surface properties of PCL materials, Figure 5D illustrates the surface qualities of PCL materials, which, as a hydrophobic material, exhibits a relatively large contact angle. After dopamine modification, the contact angle markedly diminished. With the additional introduction of copper ions, the contact angle was reduced to approximately 30° suggesting a significant improvement in the material’s hydrophilicity. The enhancement of hydrophilicity is essential for biomaterials, as it significantly improves cell adhesion and spreading on the material surface, which is beneficial for tissue regeneration and healing processes. The improved hydrophilicity facilitates the gradual release of copper ions, enabling the sustained antibacterial effect.

### 3.5 Responsiveness of pH and copper ion chelation and release

**FIGURE 6:**
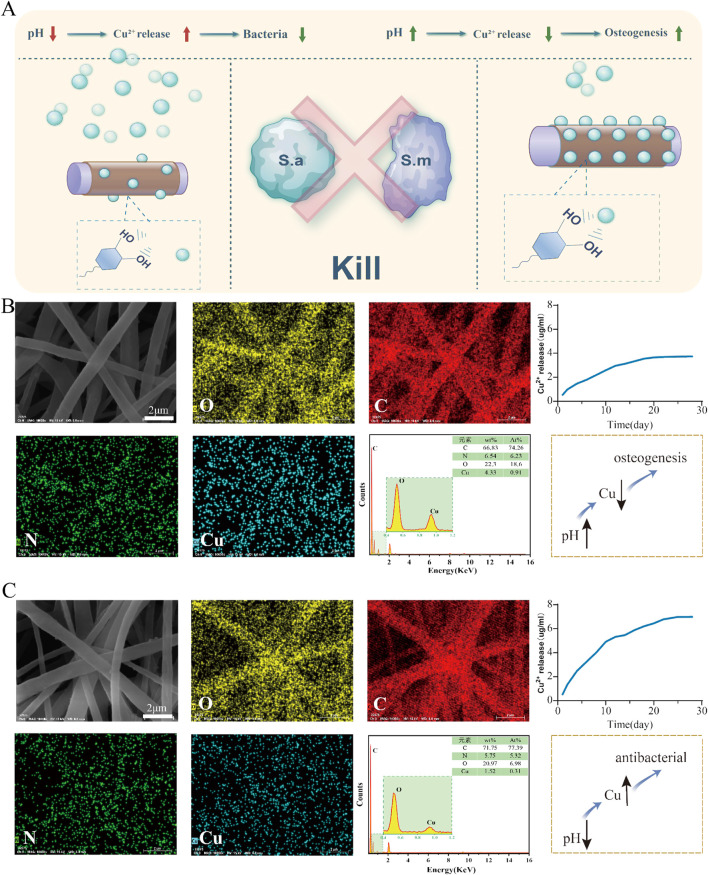
Release of copper ions from the PCL-pDA-Cu group. **(A)** Flowchart for screening the optimal copper ion concentration in the material. **(B)** SEM images and elemental distribution maps of the upper group scaffolds illustrate the distribution of oxygen, carbon, nitrogen, and copper in the fibers, and the copper ion release profile of the scaffolds in a neutral (pH = 7) environment. **(C)** SEM morphology and elemental distribution of the lower group scaffolds, demonstrating a relatively lower copper loading and the copper ion release profile of the scaffolds in an acidic (pH = 5) environment.

The experimental results Figure 6, Experimental results revealed that under different pH conditions, the electrospinning scaffolds exhibited significantly different copper ion loading and release behaviors. Under acidic conditions (pH = 5), the same material exhibited an increased copper ion release concentration. This indicates that a low pH environment markedly increases copper ion release, possibly because of the weakened binding force between PDA and copper ions under acidic conditions, hence facilitating copper ion release. This phenomenon explains why the group B exhibited stronger antibacterial activity than other groups in an acidic environment. Statistical analysis revealed that in acidic conditions, the copper ion release rate of Group B was significantly higher than that of Group A, and the antibacterial effect of Group B in this environment reached a statistically significant level (p<0.05) . In an acidic environment, the binding between PDA and copper ions may weaken, resulting in accelerated copper ion release. This characteristic is especially crucial for antibacterial efficacy, as inflamed sites often exhibit lower pH values. The rapid release of copper ions effectively inhibit bacterial proliferation; however, in a neutral environment, copper ions are gradually released, facilitating osteoblast proliferation and differentiation (Kim et al., 2023). This meticulously regulated scaffold design facilitates the controlled release or slow release of copper ions based on the environmental pH, thereby attaining an optimal balance between antibacterial effects and osteogenesis. Specifically, in inflammatory or infected conditions, the scaffold can rapidly release copper ions to produce antibacterial effects, while in neutral environments, it can gradual release, promoting osteoblast growth and differentiation. This feature gives the electrospinning scaffold excellent adaptability to many biological environments, making it highly promising for bone tissue repair and anti-infection treatment. The pH-responsive characteristic allows this scaffold to effectively adapt to dynamic biological environments, achieving a balance between antibacterial and bone regrowth functions and providing a potential solution for bone tissue repair and infection prevention.

### 3.6 Influence of the scaffold on the biological characteristics of MC3T3-E1 cells

**FIGURE 7:**
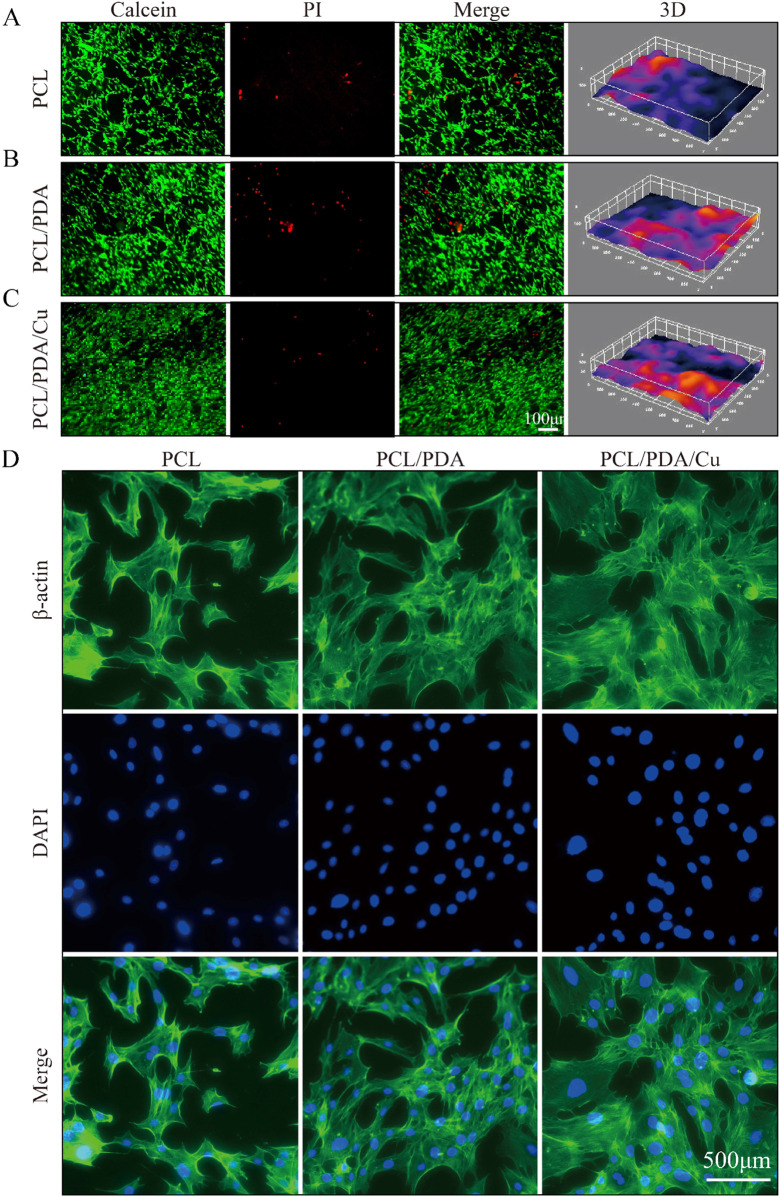
Biocompatibility analysis of different scaffold groups (Ctrl, PCL, PCL/PDA, PCL/PDA/Cu). **(A)** Live/dead cell staining results: green fluorescence (Calcein-AM) indicates live cells, **(B)** Red fluorescence (PI) indicates dead cells. **(C)** Merge represents the combined image. **(D)** F-actin staining (red) and nuclear staining (blue) of MC3T3-E1 cells on different scaffold groups (Ctrl, PCL, PCL/PDA, PCL/PDA/Cu), indicating changes in cell morphology and pseudopod structures.

Cell skeleton staining results (Figure 7D) revealed that the chemical modification of the electrospinning scaffold surface significantly affects the morphology and spreading of MC3T3-E1 cells. In the control and the unmodified PCL scaffold groups, the cell morphology was relatively dispersed; the cytoskeleton architecture was underdeveloped, and the number of filamentous pseudopodia was small, displaying limited spreading ability. This suggests that under these conditions, the scaffold surface fails to offer an ideal microenvironment for cell adhesion and expansion (Xu Y. et al., 2023). However, in the dopamine-modified scaffold (PCL/PDA) group and the copper-ion-loaded scaffold (PCL/PDA/Cu) group, the cell cytoskeleton exhibited a markedly developed structure, with an increased number of pseudopodia that were distinctly extended and improved intercellular connections. This cytoskeletal reorganization phenomenon indicates that the dopamine coating markedly improves the cellular compatibility of the scaffold surface, facilitating cell adhesion, spreading, and cytoskeletal reconstruction. Statistical analysis revealed that the cytoskeletal development in PCL/PDA and PCL/PDA/Cu groups was significantly higher than that in control and PCL groups (p<0.05) , further confirming the effectiveness of dopamine modification and copper ion loading in promoting cell morphology changes and cytoskeletal reconstruction (Li et al., 2023).

In the live/dead cell staining experiment Figures 7A–C, the effects of different scaffolds on cell viability were further verified. Calcein-AM and PI staining revealed that on the dopamine-modified scaffold (PCL/PDA) and the copper-ion-loaded scaffold (PCL/PDA/Cu), the number of live cells was significantly increased; however, the proportion of dead cells remained low. This result indicates that the synergistic effect of dopamine modification and copper ions improve cell adhesion and provide a favorable environment for cell proliferation and survival. The introduction of copper ions, despite their antibacterial function, did not exhibit significant cytotoxicity. Statistical analysis revealed that the cell survival rate in the PCL/PDA/Cu group was significantly higher than that in PCL and the control groups (p<0.01) , indicating that copper ions do not have significant toxic effects on cell viability and their antibacterial properties do not hinder cell growth (Zhao et al., 2024).

The CCK-8 cell proliferation assay revealed that the dopamine-modified and copper-ion-loaded scaffold (PCL/PDA/Cu) demonstrated excellent performance regarding cell proliferation rate and cell viability, further demonstrating its potential applications in biocompatibility, antibacterial performance, and bone regrowth induction. Developing the PCL/PDA/Cu scaffold has successfully achieved functional modification of the scaffold surface, effectively promoting the growth of osteoblasts while inhibiting bacterial growth. Statistical analysis revealed that the PCL/PDA/Cu group performed significantly better than the PCL group in enhancing cell proliferation and inhibiting bacterial growth (p<0.05) . This advanced regulatory scaffold design is applicable in bone tissue engineering and provides a feasible solution for the future development of personalized and high-performance bone defect repair materials.

### 3.7 *In Vitro* bone regrowth performance characterization

**FIGURE 8:**
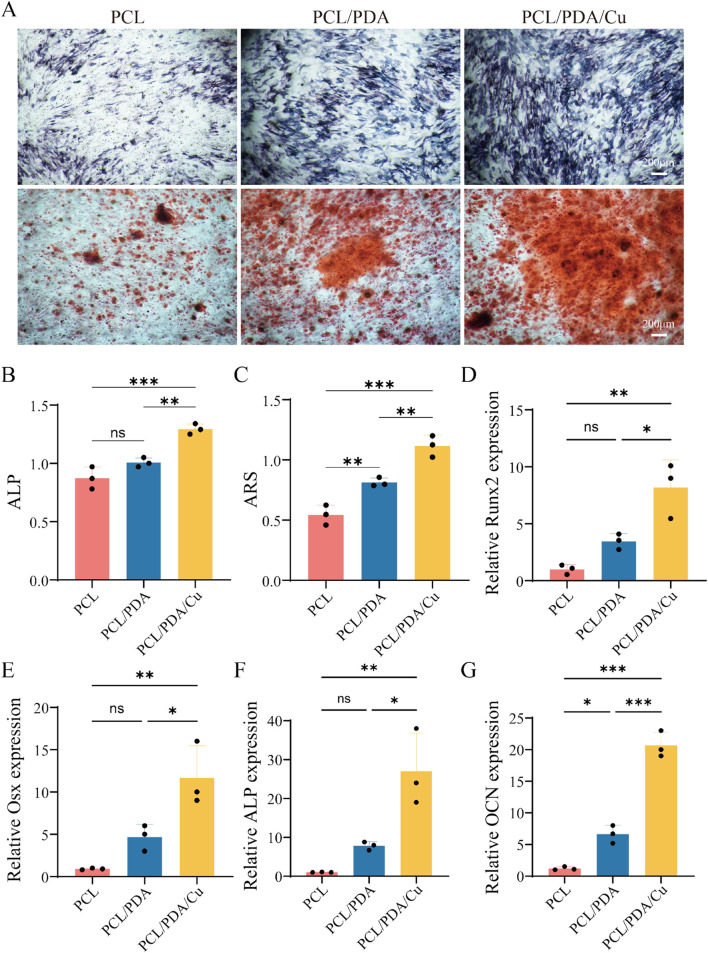
Effects of different scaffold materials on the osteogenic differentiation of MC3T3-E1 cells. **(A)** ALP and ARS. **(B)** Semi-quantitative analysis of ALP staining of MC3T3-E1 cells on different scaffold materials. **(C)** Semi-quantitative analysis of ARS of MC3T3-E1 cells on different scaffold materials. **(D–G)** Detection of osteogenesis-related genes [Runx2, Osterix (Osx), ALP, and Osteocalcin (OCN)] in MC3T3-E1 cells.

ALP and alizarin red staining are essential detection methods for evaluating osteoblasts at various differentiation stages. ALP staining is utilized to assess the activity level of early osteoblast differentiation. The extent and intensity of the blue area after staining (dark blue or blue-purple) directly reflect the expression of alkaline phosphatase, thereby evaluating bone regrowth potential. Figure 8 illustrates the ALP staining results of cells on different scaffold materials, including PCL, PCL/PDA, and PCL/PDA/Cu. The results indicate that compared with unmodified PCL, the blue staining range and color depth of PCL/PDA and PCL/PDA/Cu scaffold groups were significantly increased, indicating that dopamine modification and the introduction of copper ions markedly enhanced ALP activity, thus facilitating early osteoblast differentiation. Alizarin red staining is employed to assess the late differentiation effect and mineralization level of osteoblasts. ARS staining solution forms orange-red precipitates through its binding with calcium salts. The number and depth of orange-red areas reflect the formation of calcium nodules and are important indicators for evaluating the mineralization ability of osteoblasts. Herein, it can be visually observed through alizarin red staining (Figure 8) that there are significant differences in calcium nodule formation among different scaffold groups. PCL/PDA and PCL/PDA/Cu scaffold groups exhibited enhanced formation of deeper orange-red calcium nodules, signifying that these modified scaffolds substantially promote the mineralization and maturation of osteoblasts. ALP staining combined with alizarin red staining reveals the importance of the synergistic effect of dopamine coating and copper ions in the bone regenaration process (Zhang et al., 2023). Dopamine modification (PDA) significantly enhanced ALP activity during the initial phase of bone regrowth, facilitating the proliferation and differentiation of osteoblasts; however, the introduction of copper further improved the bone regrowth effect, particularly in the mineralization stage (Zha et al., 2024). The PCL/PDA/Cu scaffold exhibited superior efficacy in promoting the early activity and late mineralization of osteoblasts, hence demonstrating its potential to enhancing biocompatibility and promote bone regeneration. These findings establish a scientific basis for the development of multifunctional electrospinning scaffold materials for bone defect repair and indicate the extensive application potential of this scaffold in bone tissue engineering.

The PCR analysis (Figure 8) revealed that the three groups of materials, PCL, PCL/PDA, and PCL/PDA/Cu, exhibited significant differences in bone regrowth genes expression. Four key bone regrowth genes (Runx2, Osterix, ALP, and OCN) exhibited different transcriptional levels among the different material groups. In the PCL group, the expression levels of the four genes were relatively low,indicating that pure PCL material exhibited a weak bone regrowth induction effect on osteoblasts. However, the expression of bone regrowth genes, including Runx2, Osterix, and ALP significantly increased in the PCL/PDA group. This indicates that PDA effectively promotes the transcription of bone regrowth genes through its excellent hydrophilic and bioactive surface properties. This may be attributed to PDA’s ability to enhance cell-substrate interaction, thereby activating bone growth-related signaling pathways. In the PCL/PDA/Cu group, the expression levels of all four genes further increased, which were significantly higher than those in the PCL/PDA group. This indicates that the introduction of copper ions significantly promoted the bone regrowth process. Copper ions regulate bone formation by influencing cell metabolism, stimulating cell proliferation and differentiation, and promoting the expression of bone regrowth genes, thereby further augmenting bone regrowth potential. In the quantitative ALP test, the ALP activity in PCL/PDA and PCL/PDA/Cu groups was significantly higher than that in the pure PCL group, particularly in the PCL/PDA/Cu group, where the ALP activity reached the highest level, further verifying the promoting effect of copper on bone regrowth enzyme activity. Copper ions are essential in bone repair, and their mechanism of promoting osteogenesis may be related to their regulation of signal transduction pathways through cell receptors and the enhancement of ECM deposition and mineralization. The quantitative ARS analysis revealed that the number and area of mineralized nodules in the PCL/PDA/Cu group were much higher than those in PCL and PCL/PDA groups, further confirming the promoting effect of copper ions on the bone mineralization process (Zha et al., 2024). The number of mineralized nodules in the PCL/PDA group was increased compared to that of the pure PCL group, indicating that the introduction of PDA exhibited a certain promoting effect on the mineralization process. PCL/PDA and PCL/PDA/Cu composite materials significantly enhanced the expression of bone regrowth genes and bone regrowth function, particularly after the introduction of copper ions; the bone regrowth-promoting effect was further strengthened. These results indicated that PCL/PDA/Cu composite materials have broad application prospects in bone repair and regeneration, particularly in the treatment of bone defects and bone tissue engineering.

### 3.8 Antibacterial characteristics

**FIGURE 9:**
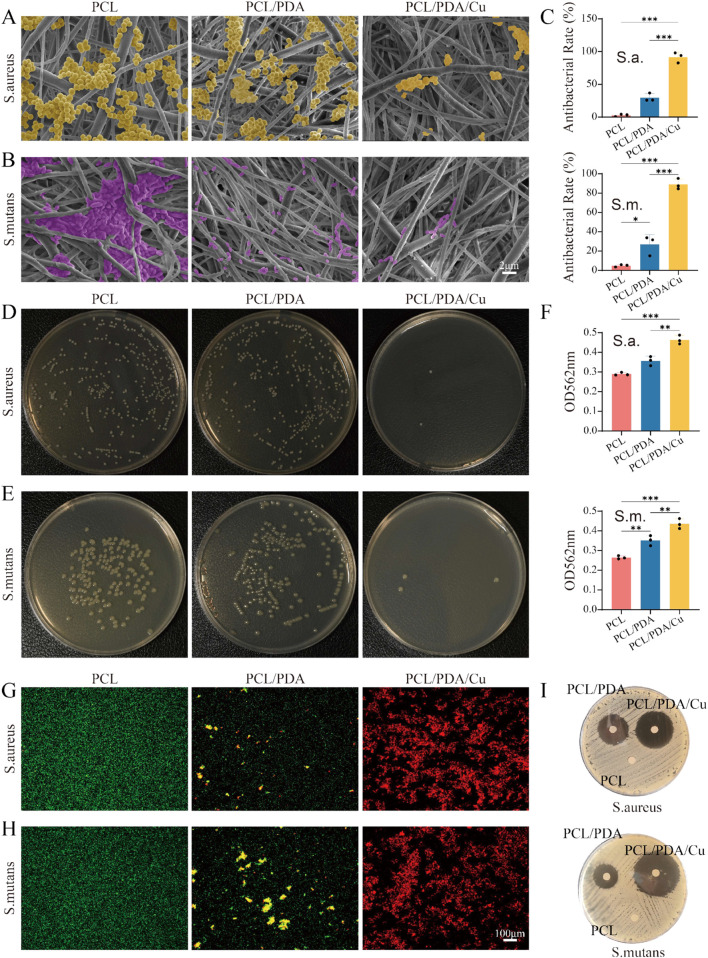
Antibacterial rate. **(A,B)** SEM images of *Staphylococcus aureus* (*S. aureus*) and *Streptococcus* mutans (S. mutans) on three different scaffold surfaces (PCL, PCL/PDA, PCL/PDA/Cu), with bacteria labeled in yellow and purple, respectively. The results demonstrate the effects of different scaffold materials on bacterial adhesion and growth. **(C–E)** Plate counting results of *S. aureus* and S. mutans after culturing on PCL, PCL/PDA, and PCL/PDA/Cu scaffolds. **(F)** Absorbance of two bacterial lysates at 562 nm was measured using the BCA method. **(G,H)** Live/dead bacterial staining results of *S. aureus* and S. mutans after culturing on PCL, PCL/PDA, and PCL/PDA/Cu scaffolds. **(I)** Inhibition zone results of *S. aureus* and S. mutans after culturing on PCL, PCL/PDA, and PCL/PDA/Cu scaffolds.

In the complex oral environment, preventing postoperative infections is one of the key challenges in the formulation of bone tissue engineering materials. Herein, electrospinning PCL scaffolds were subjected to dopamine modification (PCL/PDA) and further copper ion impregnation (PCL/PDA/Cu) to enhance their antibacterial properties, facilitating better clinical applications. The results from SEM images (Figure 9) demonstrated the significant effect of different scaffold materials on bacterial adhesion. The unmodified PCL scaffold surface demonstrates significant adherence to *S. aureus* and S. mutans, indicating poor intrinsic antibacterial properties of PCL. However, after dopamine modification (PCL/PDA), the quantity of bacterial adhesion decreased, particularly with a significant reduction in the number of *S. aureus*, demonstrating that dopamine inhibits bacterial adhesion to some extent (Mutalik et al., 2022). Conversely, the copper-loaded dopamine-modified scaffold (PCL/PDA/Cu) exhibited the most significant antibacterial effect, significantly reducing *S. aureus* and S. mutans, with almost no visible bacterial adhesion. This remarkable reduction is likely attributed to the antibacterial properties of copper ions, which can disrupt bacterial cell membranes, inhibiting bacterial growth and proliferation. The plate count experiment further confirmed the SEM observations. In the untreated PCL scaffold group, a significant quantity of bacterial colonies were observed on the plate, indicating that the PCL scaffold did not inhibit bacterial growth. After dopamine modification (PCL/PDA), the number of bacterial colonies decreased, especially for *S. aureus*, hence confirming the inhibitory effect of dopamine on bacterial adhesion. In the PCL/PDA/Cu scaffold group, the bacterial colony count significantly decreased, with almost no visible colonies of *S. aureus* or S. mutans, indicating that copper ion loading effectively prevented bacterial growth. The antibacterial mechanism of copper ions is generally associated with cell membrane disruption and inhibition of key intracellular enzymes. Copper ions bind to proteins on the bacterial cell wall and membrane, disrupting the structural integrity of the cell and resulting in leakage of cellular contents. Copper ions can generate ROS, which further compromise bacterial membranes and DNA, inhibiting bacterial growth or resulting in bacteria death. In the PCL/PDA/Cu scaffold, the copper ion loading enhanced antibacterial performance and significantly reduced adhesion and growth of the two clinically relevant bacteria. This characteristic renders the PCL/PDA/Cu scaffold exceptionally beneficial for clinical applications, especially in oral surgeries or bone repair scenarios where infection prevention is critical.

## 4 Conclusion

This study effectively developed an electrospinning scaffold using dopamine’s metal ion chelation properties and pH responsiveness to attain a balance between antibacterial efficacy and bone regeneration performance. The specific advantages are listed as follows: (1) The material is microenvironmentally responsive, facilitating localized control of copper ion release and recovery during the infection and bone healing stages. (2) Copper ion release at low pH conditions during infection, effectively providing antibacterial effects. (3) Exceptional bone regrowth properties, supporting bone regeneration. (4) As a GBR membrane, it exhibits exceptional bone-forming and antibacterial properties. This new scaffold exhibits good biocompatibility and regulates antibacterial and bone regrowth activity in different microenvironments, exhibiting great potential for bone tissue regeneration and anti-infection applications.

## Data Availability

The original contributions presented in the study are included in the article/supplementary material, further inquiries can be directed to the corresponding author.
